# Case Report: *In Situ* Expression of a Proliferation-Inducing Ligand in Neuromyelitis Optica

**DOI:** 10.3389/fneur.2021.721877

**Published:** 2021-08-05

**Authors:** Laurie Baert, Romain Marignier, Hans P. Lassmann, Bertrand Huard

**Affiliations:** ^1^Institute for Advanced Biosciences, University Grenoble-Alpes, Grenoble, France; ^2^MIRCEM, P. Wertheimer Neurology Hospital, Lyon, France; ^3^Center for Brain Research, Medical University, Vienna, Austria

**Keywords:** neuromyelitis optica, TACI, astrocytes, B cells, APRIL (TNFSF13)

## Abstract

A proliferation inducing ligand (APRIL) mediates a key role in the generation and survival of antibody-inducing plasmocytes. Based on this, APRIL has been targeted in autoimmune diseases including multiple sclerosis (MS) and optic neuritis (ON). In MS lesions, APRIL has a new cellular target, the reactive astrocyte and mediates an immunosuppressive activity. Here, we analyzed APRIL expression in a case of neuromyelitis optica (NMO), another autoimmune neurodegenerative disease, showing selective aquaporin-4 depletion in the spinal cord, complement deposition and infiltration of polymorphonuclear cells. We analyzed by immunohistochemistry the presence of APRIL-producing cells, plasmocytes, astrocytes and the localization of secreted APRIL in a lesion from NMO. Plasmocytes were present close to APRIL-producing cells in meninges. However, our main observation was that APRIL targets reactive astrocytes in this lesion of NMO similarly to MS.

## Introduction

B cells are currently targeted with success in autoimmune diseases (1). The B-cell activation factor from the TNF family (BAFF, TNFSF13b) and a proliferation inducing ligand (APRIL, TNFSF13) are two related members from the TNF superfamily that play non-redundant role in humoral immunity (2). BAFF mostly acts on naive mature B cells, while APRIL acts on more differentiated B cells, the antibody-producing plasmocytes. These two molecules may be antagonized by the single agent atacicept, a soluble form of one of their common receptors, the transmembrane activator and CAML interactor (TACI, TNFRSF13b) (3). The completion of a recent phase II clinical trial in systemic lupus erythematosus revealed that atacicept reduces disease activity and flares with an acceptable safety profile (4). Atacicept was also tested in relapsing multiple sclerosis (MS) with the ATAMS trial (5). Consistent with a combined BAFF/APRIL blockade, the mature B-cell count and total immunoglobulins dropped in sera, indicative of an appropriate biological response. However and quite unexpectedly, an increased relapse rate was observed leading to an early trial halt. This suggested an alternative role for the BAFF/APRIL axis in the central nervous system (CNS). We recently showed that APRIL was selectively expressed in MS lesions, and induces an anti-inflammatory response by binding to reactive astrocytes (6). In an animal model of MS, we further observed disease worsening in the absence of APRIL, demonstrating an overall neuroprotective role for APRIL in the CNS. Such a new role for APRIL is a likely explanation, at least in part, for the ATAMS trial failure observed in MS.

Concomitant to ATAMS was the trial ATON testing atacicept in optic neuritis (ON) with the exclusion of NMO (7). For safety reason, ATON was also halted once the increased relapsing events were noted in ATAMS. ATON premature analysis also revealed disease worsening with an extension to a relapsing-remitting MS-like syndrome in a significant fraction of treated patients. Contrary to MS, there is the strong association of NMO with a specific humoral immune response directed against aquaporin 4 (AQP4) expressed at the surface of astrocytes (8). Thus, atacicept might have a pronounced protective effect in principle in this disease. Based on this, a new form of soluble TACI, telitacicept, is currently tested in recurrent NMO (9). We analyzed APRIL expression in lesions present in the spinal cord from a NMO patient.

## Case Report

**Figure 1:**
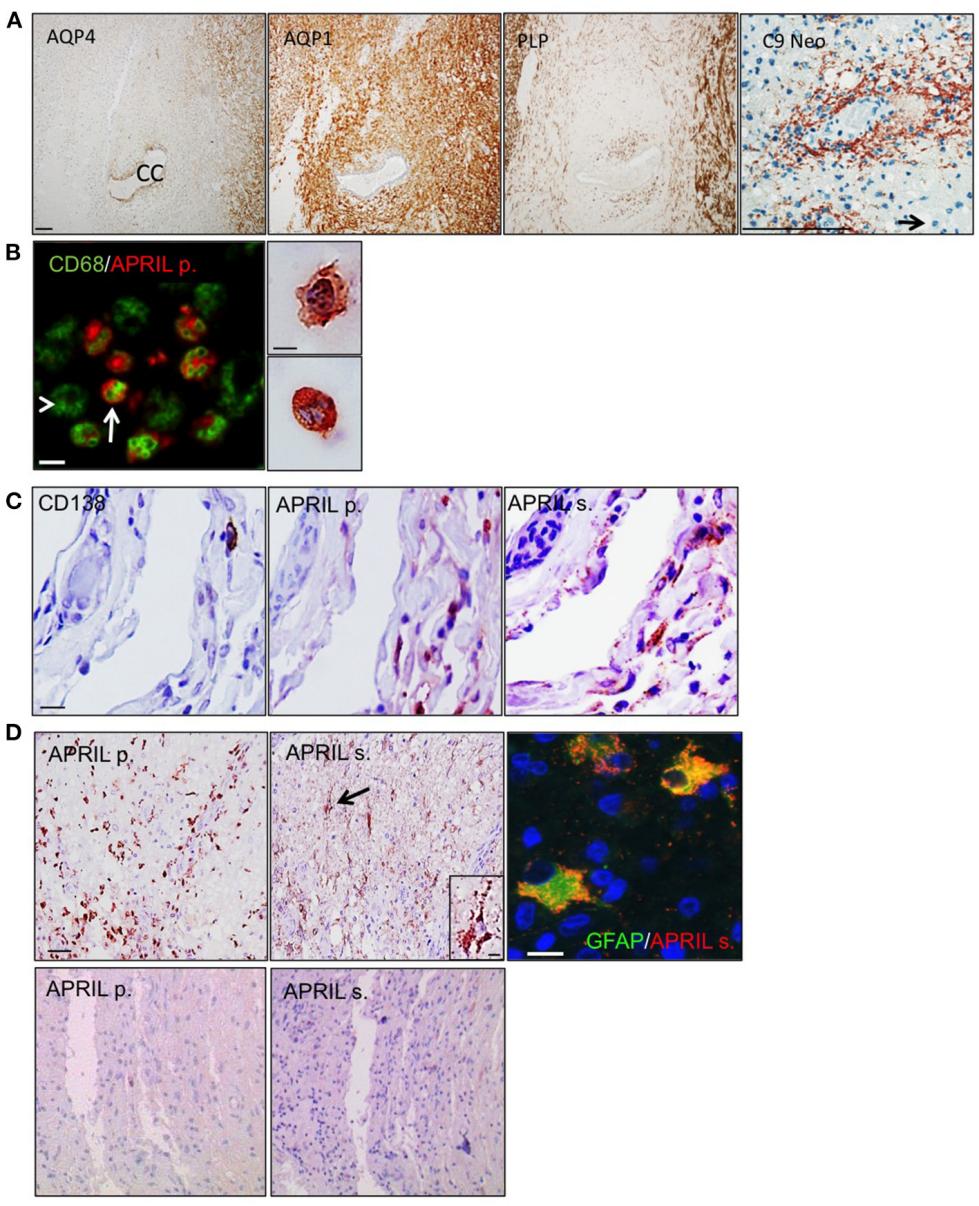
APRIL targets reactive astrocytes in NMO lesions. Serial sections of a NMO biopsy harboring an active lesion were immunostained. **(A)** shows a widespread loss of aquaporin 4 (AQP4) associated to preservation of aquaporin 1 (AQP1), and proteolipid protein (PLP)-positive myelin sheaths. A vasculocentric (rosette-like) deposition of activated complement (C9 neo) is observed in an active lesion area rich in polymorphonuclear cells (scale bar = 100 μm). The central canal (CC) of the spinal cord and a polymorphonuclear cell (arrow) are indicated. **(B)** shows merge stainings for CD68^+^ myeloid cells and APRIL-producing cells (APRIL p.) (scale bar = 10 μm). Arrow and arrowhead mark APRIL^+^ and APRIL^−^ CD68^+^ myeloid cells, respectively. Right panel shows a high magnification of APRIL-producing cells with a macrophage (top figure) and polymorphonuclear (bottom figure) morphology (scale bar = 5 μm). **(C)** shows in meninges associated to the lesion stainings for plasmocytes (CD138), APRIL p. and secreted APRIL (APRIL s.) (scale bar = 10 μm). Upper panels **(D)** show in the parenchyma stainings for APRIL p. and APRIL s. (scale bar = 100 μm). Cells binding APRIL with a morphology of astrocytes are arrowed, showed in the high magnification insert (scale bar, 5 μm), and identified by GFAP costaining (scale bar = 5 μm). Lower panels **(D)** show an area outside lesions of the spinal cord stainings for APRIL p. and APRIL s. (scale bar 100 μm).

The patient was a female of 20 years harboring the clinical criteria for the diagnosis of a definite NMO, although at the time of her death AQP4 antibody testing was not yet established. Two weeks before her death, she experienced a massive relapse of myelitis, which broadly progressed and conducted the patient into coma. She had a disease duration of four years, and died from pulmonary embolism. The patient did not receive any immunosuppressive treatment. Autopsy demonstrated extensive lesions in the spinal cord, the brain stem and also large lesions in the forebrain hemispheres, which had been described previously by Misu et al., for the case 499 (10). At the spinal cord level, typical pathological features of NMO were seen with a widespread loss of AQP4 within and around the lesions, while AQP1 reactivity was partially spared (Figure 1A). Myelin sheaths assessed by proteolipid protein (PLP) immunoreactivity were preserved indicative of an early stage active lesion. Finally, a profound rosette-like perivascular complement activation (C9 neo reactivity) combined with an infiltration of polymorphonuclear granulocytes was also evidenced. To analyze APRIL expression, we used the pair of antibodies identifying APRIL-producing cells and secreted APRIL in tissues (11). APRIL-producing cells were part of the CD68^+^ myeloid cell pool (Figure 1B). We identified these cells by morphology as macrophages/microglia and polymorphonuclear cells. The common APRIL target cells, CD138^+^ plasmocytes, were sparsely distributed within meninges adjacent to lesions (Figure 1C). They were in close vicinity to APRIL-producing cells. We could also detect tissue retention of secreted APRIL in this area. This indicates that APRIL may sustain the survival of plasmocytes present in the CNS of NMO patients. In the parenchyma, APRIL-producing cells were highly abundant, representing as much as 50% of the total cellularity in some area (Figure 1D upper panels). There, plasmocytes were absent, but secreted APRIL targeted another cell type identified by staining for the glial fibrillary acidic protein (GFAP) as reactive astrocytes. Accumulation of secreted APRIL in astrocytes was quite high, filling up the entire cytoplasmic space of the cells. APRIL-producing cells and secreted APRIL were absent outside lesions from this spinal cord (Figure 1D lower panels). Hence, APRIL also targets astrocytes in NMO lesions.

## Discussion

Based on this study, APRIL may provide a favorable environment for plasmocytes in NMO lesions, due to the close vicinity between APRIL-producing cells and plasmocytes within meninges. An APRIL-targeting agent may reduce the number of CNS-infiltrated plasmocytes in NMO. However, the targeting of reactive astrocytes by secreted APRIL also indicates that APRIL may induce an anti-inflammatory response in NMO lesions. Taken together, our data highlight that trials targeting APRIL in the CNS have to be conducted with extreme cautiousness.

## Data Availability Statement

The original contributions presented in the study are included in the article/supplementary material, further inquiries can be directed to the corresponding author.

## Ethics Statement

The studies involving human participants were reviewed and approved by ethics comittee, university of Vienna, Austria. The patients/participants provided their written informed consent to participate in this study.

## Author Contributions

LB performed experimentation. HL provided the case. HL and RM analyzed the data. BH designed the study, analyzed the data, and wrote the manuscript. All authors contributed to the article and approved the submitted version.

## Conflict of Interest

BH has received speaker bureau honoraria from Merck Serono who is developing atacicept. The remaining authors declare that the research was conducted in the absence of any commercial or financial relationships that could be construed as a potential conflict of interest.

## Publisher's Note

All claims expressed in this article are solely those of the authors and do not necessarily represent those of their affiliated organizations, or those of the publisher, the editors and the reviewers. Any product that may be evaluated in this article, or claim that may be made by its manufacturer, is not guaranteed or endorsed by the publisher.
